# Crosstalk between transposase subunits during cleavage of the *mariner* transposon

**DOI:** 10.1093/nar/gku172

**Published:** 2014-03-12

**Authors:** Corentin Claeys Bouuaert, Neil Walker, Danxu Liu, Ronald Chalmers

**Affiliations:** School of Life Sciences, University of Nottingham, Queen's Medical Centre, Nottingham NG7 2UH, UK

## Abstract

*Mariner* transposition is a complex reaction that involves three recombination sites and six strand breaking and joining reactions. This requires precise spatial and temporal coordination between the different components to ensure a productive outcome and minimize genomic instability. We have investigated how the cleavage events are orchestrated within the *mariner* transpososome. We find that cleavage of the non-transferred strand is completed at both transposon ends before the transferred strand is cleaved at either end. By introducing transposon-end mutations that interfere with cleavage, but leave transpososome assembly unaffected, we demonstrate that a structural transition preceding transferred strand cleavage is coordinated between the two halves of the transpososome. Since *mariner* lacks the DNA hairpin intermediate, this transition probably reflects a reorganization of the transpososome to allow the access of different monomers onto the second pair of strands, or the relocation of the DNA within the same active site between two successive hydrolysis events. Communication between transposase subunits also provides a failsafe mechanism that restricts the generation of potentially deleterious double-strand breaks at isolated sites. Finally, we identify transposase mutants that reveal that the conserved WVPHEL motif provides a structural determinant of the coordination mechanism.

## INTRODUCTION

**Figure 1.**
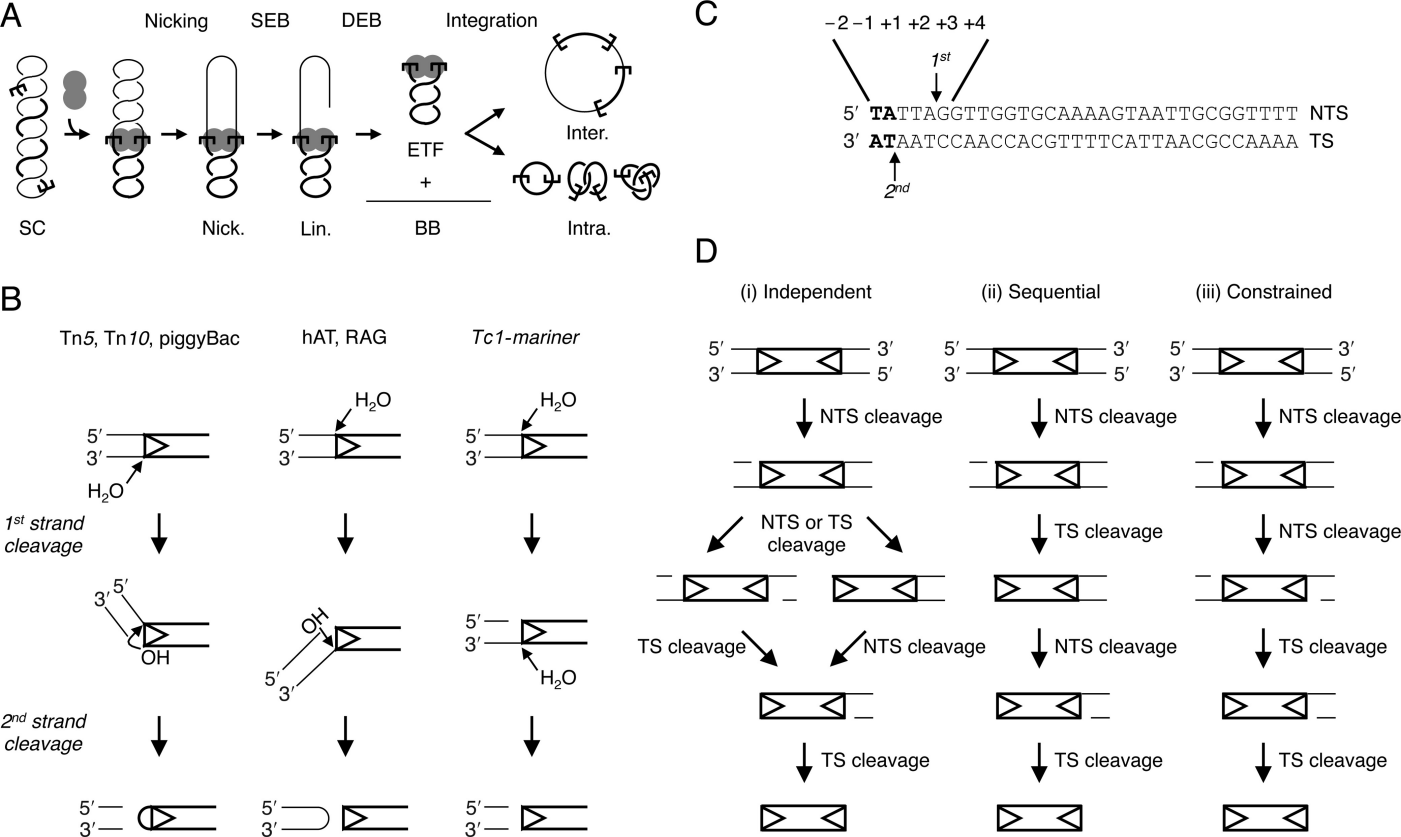
Cut-and-paste transposition and the mechanisms of double-strand cleavage at transposon ends. (**A**) A cartoon of *mariner* transposition with a supercoiled (SC) substrate as a transposon donor. The transpososome assembles two transposon ends and a transposase dimer. Non-transferred strand (NTS) nicking generates an open circular product (Nick.). Transferred strand (TS) nicking at one end yields the linear SEB product (Lin.). A similar set of nicks at the other transposon end yields the two double-end break (DEB) products, which are the plasmid backbone (BB) plus the excised transposon fragment (ETF). Examples of inter- and intra-molecular integration products are illustrated (8,14). (**B**) Three mechanisms for DSB formation in DNA transposition (17). Single transposon ends are represented as double-stranded DNA. Transposon DNA is shown as thick lines, flanking DNA as thin lines, transposon ends as arrowheads. Tn*5* and Tn*10* in bacteria and piggyBac in eukaryotes cleave the TS (bottom strand) first. The resulting 3′-OH is used as a nucleophile to attack the opposite DNA strand, which generates a hairpin at the transposon end. Eukaryotic hAT elements and RAG proteins use a reverse hairpin intermediate. The NTS (top strand) is cleaved first and the resulting 3′-OH attacks the opposite strand to give a hairpin on the flanking DNA. Members of the *Tc1-mariner* superfamily do not use a hairpin intermediate. The NTS is cleaved first then the second strand break occurs by a second hydrolysis reaction. (**C**) Sequence and cleavage sites of the *Hsmar1* transposon end. The NTS is cleaved preferentially 3-bp within the transposon ends. The TS is then cleaved exactly at the intersection between the transposon and the flanking DNA, as indicated by the arrows (24,29). *Tc1-mariner* elements are also flanked by symmetrical TA dinucleotides, which result from the duplication of the target site upon integration (26). Nucleotide numbering of the NTS is indicated. (**D**) Three possible models for the order of catalytic events during cleavage of the *mariner* transposon. The NTS is always cleaved before the TS at a given transposon end (panel B). Cleavage events at the two ends can either be (i) independent, (ii) sequential, with one end always cleaved before the other, or (iii) constrained, with both NTS always cleaved before cleavage of the first TS.

Transposons are genetic elements that mobilize and amplify within a host genome. Although they are generally detrimental, and often therefore considered as molecular parasites, they are also important evolutionary forces (1). Cut-and-paste transposition is catalysed by the element-encoded transposase. To excise the transposon from the donor site, the transposase generates a double-strand break (DSB) at both ends of the element. This is followed by integration of the transposon in a new target site (Figure 1A). Since the transposition reaction represents a danger to the host and the transposon itself, regulation is important to avoid deleterious events. These include the formation of DSBs at an isolated site, the failure of the transposon to reintegrate or other more complex genome rearrangements such as those that arise from single-end strand transfer (e.g. (2)).

Coordination of the catalytic steps of the reaction between the two transposon ends will help to avoid deleterious events. One way in which this can be achieved is by the *trans* architecture of the transpososome, in which a transposase subunit bound to one transposon end performs catalysis at the opposite end (3,4). In the bacterial elements Tn*10* and Tn*5*, where the free transposase is a monomer, this is an effective strategy because dimerization couples transposon end synapsis to catalysis. During catalysis, Tn*10* transposase subunits also crosscommunicate. This was first suggested by genetic studies in which a wild-type partner rescued catalysis at a transposon end that was mutated in the vicinity of the cleavage site (5). Subsequent biochemical studies showed that the communication extends to the resolution of the hairpin intermediate, which is required to complete the cleavage reaction (6,7).

In *mariner* transposition and V(D)J recombination the transposases/recombinases are dimers in solution (8,9,10). In principle, this allows the possibility of uncoordinated cleavage events if an active site has access to the DNA before synapsis. This appears to be the case during V(D)J recombination, where the RAG recombinases can nick an unsynapsed recombination signal sequence (11). In this case, coordination is then established before cleavage of the second strand, which depends on synapsis and prior nicking of the partner (12,13). In contrast, recruitment of the second transposon end into the *mariner* transpososome is needed to support a significant rate of catalysis (14,15). This shows that signals must pass between the subunits during transpososome assembly.

The transposon ends, which provide the transposase-binding sites, are asymmetrical sequences (i.e. not palindromic). This presents a particular problem for a double-strand nuclease in that an elaborate strategy seems to be required to deal with the two DNA strands, which are of opposite polarity (16). This contrasts notably with the familiar type IIP restriction endonucleases where two identical subunits recognize identical half-sites of a palindromic sequence and perform identical single-strand nicks. Several of the DDE/D family of transposases deal with the two strands using a DNA hairpin intermediate (17–21). This mechanism comes in two flavors, depending on whether the ‘top’ or ‘bottom’ strand of the transposon end is cleaved first (Figure 1B). In either case, the first nick exposes a 3′-OH, which acts as the nucleophile in a direct transesterification reaction that attacks the opposite strand. If the hairpin is on the end of the transposon, it must be resolved before the integration step.

In contrast, the *mariner Mos1* transposase, which is also a member of the DDE/D family, does not conform to the hairpin paradigm (22). The simplest alternative is that the strands are nicked by sequential hydrolysis reactions. However, if this is the case it begs the question of how the active site is able to accommodate strands of opposite polarity. One possibility that has been suggested is that the non-transferred strand (NTS) and the transferred strand (TS) are cleaved by different transposase subunits in *cis* and *trans*, respectively (4,22). Since the NTS is always cleaved before the TS, this would presumably require that both NTS events would have to take place before either of the TS events.

To illuminate this issue, we have investigated the ways in which the catalytic steps at each end and on each strand are dependent on each other. We define the order of catalytic events within the transpososome and show that there is crosstalk between the two halves of the transpososome during transposon cleavage. Specifically, we demonstrate that a structural transition, which takes place between NTS and TS cleavage, is coordinated between the two sides of the complex. This probably reflects the mechanism for cleaving two strands of opposite polarity. In addition, it serves to suppress the generation of DSBs in the event that one of the transposon ends has acquired an inactivating mutation. Finally, we also show that the signals are transduced by a long unstructured loop that links the catalytic core of one subunit with the conserved WVPHEL ‘linker’ motif of the other.

## MATERIALS AND METHODS

DNA-modifying enzymes were from New England Biolabs. Oligonucleotides were from Sigma. All cloned polymerase chain reaction products were confirmed by DNA sequencing.

### Protein purification and *in vitro* transposition assay

The reconstructed ancestral *Hsmar1* transposase was expressed, purified and assayed as described previously (8,14). Briefly, wild-type transposase was expressed as a maltose-binding protein fusion from pRC880. Transposition assays with supercoiled transposon donors contained 6.7 nM of plasmid substrate and 20 nM transposase in 20 mM Tris-HCl pH 8, 100 mM NaCl, 2 mM DTT, 2.5 mM MgCl_2_ and 10% glycerol. Transposition reactions were incubated at 37ºC and analyzed by loading 500 ng of the DNA on each lane of a Tris-borate-EDTA (TBE)-buffered 1.1% agarose gel. After electrophoresis, the gel was stained with ethidium bromide, destained in water and photographed. For strand cleavage analyses of reactions using a supercoiled substrate, the products of transposition reactions were digested with the BsaHI restriction endonuclease and 3′-labeled with α-^32^P-dCTP and the Klenow enzyme or dephosphorylated with Antarctic phosphatase and 5′-labeled with γ-^32^P-ATP and the polynucleotide kinase (PNK) enzyme. Products were separated on a 1.5% alkaline agarose gel (50 mM NaOH, 1 mM EDTA), the gel was dried and recorded on a Fuji phosphorimager.

### Plasmid substrates

The standard supercoiled substrate was pRC650, which encodes a mini-transposon with 30 bp *Hsmar1* transposon ends and flanking TA dinucleotides. Transposon donors with two symmetrical mutations at the transposon ends were pRC1317 (−1T), pRC1318 (−1C), pRC1319 (−1G), pRC1320 (−2A), pRC1321 (−2C), pRC1322 (−2G), pRC1323 (+1A), pRC1324 (+1C), pRC1325 (+1G), pRC1326 (+2A), pRC1327 (+2C), pRC1328 (+2G), pRC1329 (+3T), pRC1330 (+3C), pRC1331 (+3G), pRC1362 (+4A), pRC1363 (+4T), pRC1364 (+4G) and pRC1341 (5G × 5G). Transposon donors with one wild-type (WT) and one mutant transposon end were pRC1342 (WT × 5G), pRC1370A (WT × −1T), pRC1370B (−1T × WT), pRC1380 (WT × +1A), pRC1381 (WT × +1C) and pRC1382 (WT × +1G). The nomenclature for the mutant substrates is as follows: negative numberings are for positions within the flanking DNA and positive numberings are for positions within the transposon end, as shown in Figure 1C. A mutation is named after the nucleotide of the NTS. In the 5G mutation five consecutive GC base pairs replace the nucleotides located from 2 bp within the flanking DNA to 3 bp within the transposon end.

## RESULTS

*Hsmar1* entered the human genome about 50 million years ago. It remained active for about 5 million years before the invasion succumbed to genetic drift (15,23). Here we use the transposase of the ancestral founding element, which was reconstructed by phylogenetic analysis of about 200 inactive copies present in the human genome (24). We elected to work with this enzyme because it is biochemically well behaved and lacks the non-specific nuclease activity associated with *Mos1* ((8,14,25,26) and Takac M. and RC, unpublished). We set out to characterize the mechanism of cleavage within the *Hsmar1* transpososome. Several important features of transposon end cleavage have already been established for *Mos1* and the correct interpretation of the current work depends on knowing whether *Hsmar1* uses the same mechanism. Specifically, the *Mos1* experiments have defined the mechanism of double-strand cleavage at a given end (22). The DSB proceeds through sequential hydrolysis reactions, not via a hairpin intermediate. The NTS is cleaved first, usually 3 bp within the transposon end, followed by cleavage of the TS, precisely at the junction between the transposon and the flanking DNA (Figure 1B and C). We recapitulated the relevant *Mos1* experiments and found that the mechanism of *Hsmar1* is indeed the same (Supplementary text and Figure S1).

### Both NTS are cleaved within the single-end-break intermediate

The order of events at a given end being defined, we went on to address how the cleavage events at opposite ends of the transposon may be related to each other. There are only three ways in which the two double-strand cleavage events may be conducted: (i) a model in which the NTS and TS nicking at each end are independent of each other, (ii) a sequential DSB model in which the break at one end is completed before it is initiated at the other or (iii) a constrained model in which both NTS are nicked before the first TS is cleaved (Figure 1D).

The order in which the strand cleavage events are conducted at the two transposon ends can be established by examining the single-end-break (SEB) intermediate (see Figure 1A). This species has already undergone NTS and TS cleavage on one end. The presence or absence of single-strand nicks at the other end can help to distinguish between the three models. Because the NTS is always cleaved first at a given end, any single-strand nick detected will necessarily be present on the NTS. If the NTS of the uncleaved transposon end is always nicked, it would support the constrained-order model.

**Figure 2.**
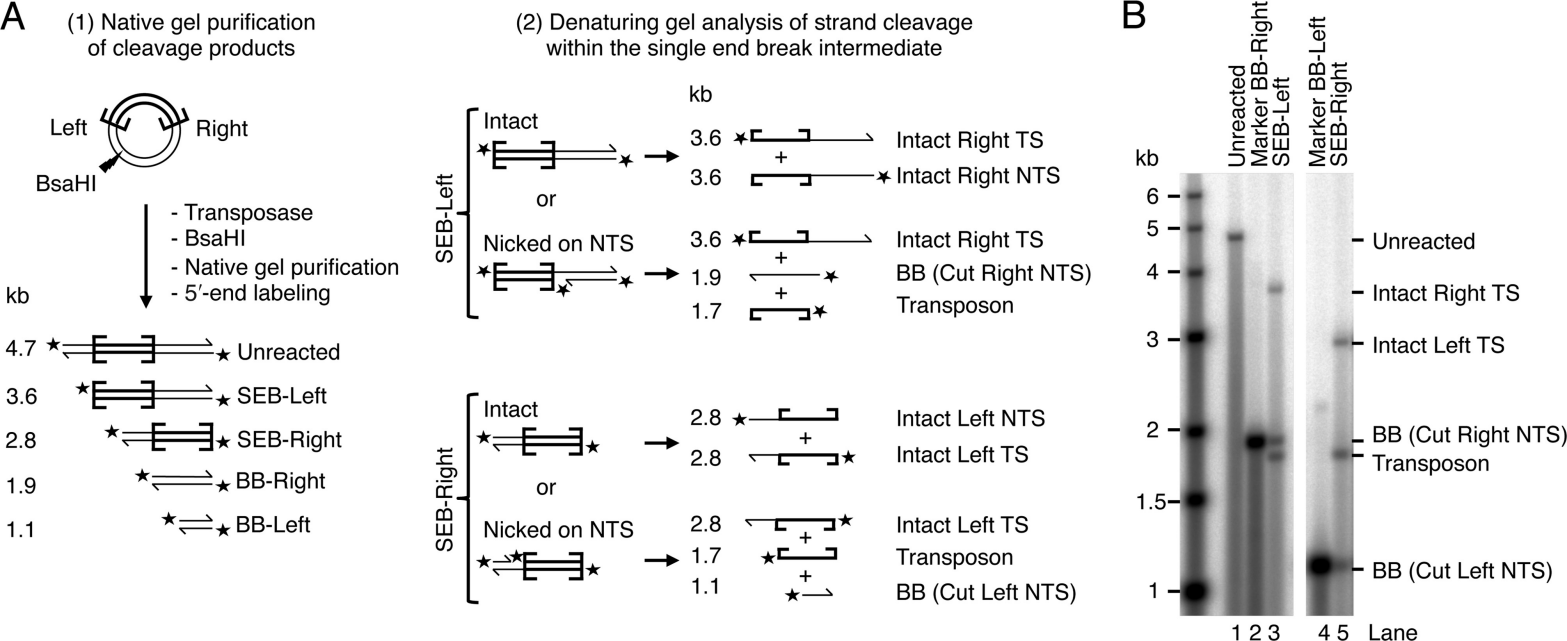
Both NTS are cleaved in the SEB product. (**A**) The products of a transposition reaction using a supercoiled plasmid substrate (pRC650) were digested within the plasmid backbone with the restriction enzyme BsaHI, dephosphorylated and 5′-labeled with γ-^32^P-ATP and PNK. Products were separated on a native agarose gel and purified. The SEB products were tested for the presence or absence of single-strand nicks at the opposite end. This assay cannot distinguish if a single-strand nick is located on the NTS or on the TS. However, since the NTS is always cleaved first at a given transposon end ((22) and Supplementary Figure S1), a single-strand nick must be on the NTS. The unreacted substrate and backbone (BB) fragments were also gel purified and used as molecular markers. (**B**) The gel-purified SEB products (SEB-Right and SEB-Left), the unreacted full-length substrate and the backbone fragments (BB-Right and BB-Left) were analyzed by denaturing agarose gel electrophoresis. The gel was dried and recorded by autoradiography.

**Figure 3.**
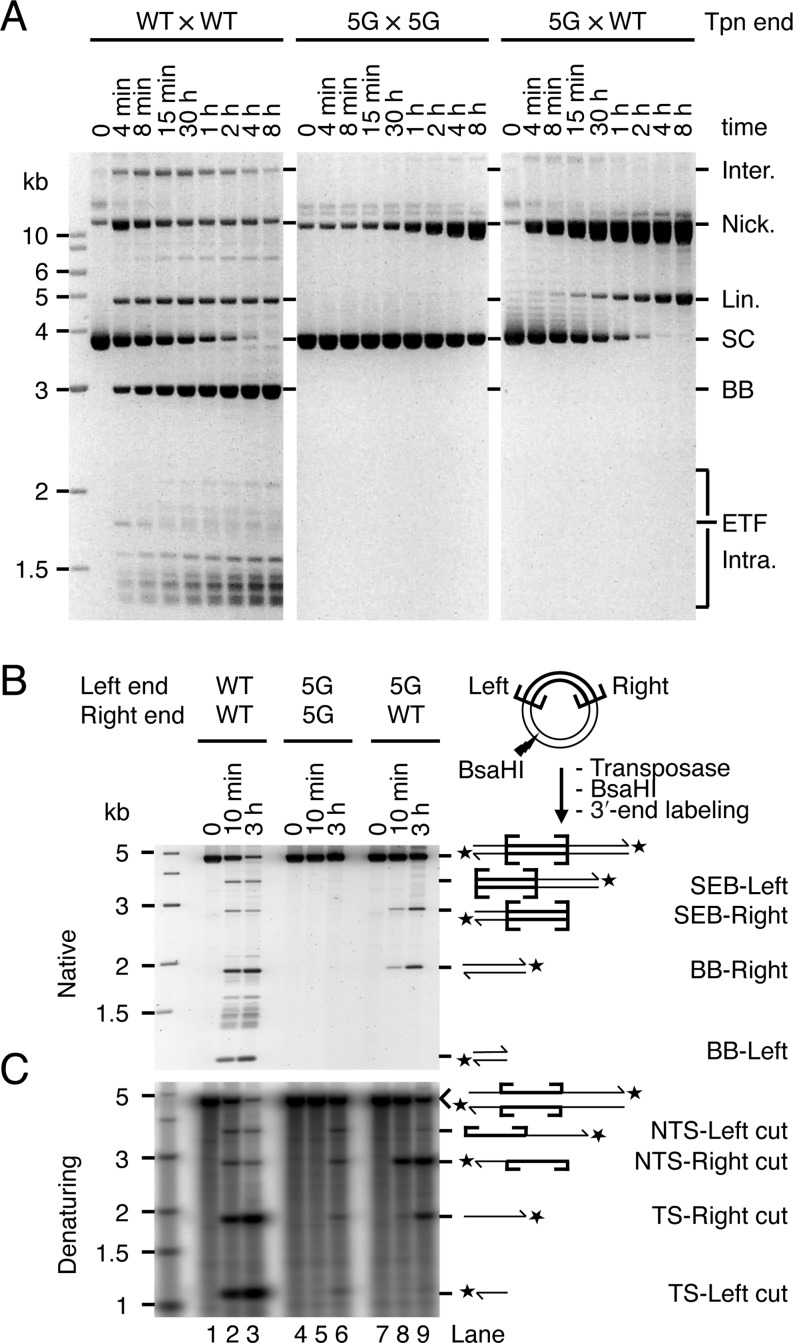
The two transposon ends are not independent during catalysis. (**A**) The kinetics of transposition reactions with a supercoiled substrate that carried two wild-type transposon (Tpn) ends (WT × WT), two 5G mutant transposon ends (5G × 5G) or a 5G mutant and a wild-type end (5G × WT) were analyzed by native agarose gel electrophoresis. The intermediates and products of these reactions are as illustrated in Figure 1A, except that the samples were deproteinated before loading the gel. The identity of these products has been determined previously by restriction digestion analysis, one- and two-dimensional gel electrophoresis, and DNA sequencing (8,14,26). The 5G mutant has an array of five guanine nucleotides spanning from position −2 to +3 on the NTS (see numbering in Figure 1C). (**B, C**) The products of transposition reactions with the substrates used in part A were digested with the restriction enzyme BsaHI, 3′-labeled with α-^32^P-dCTP and the Klenow enzyme and analyzed by native (B) and denaturing (C) agarose gel electrophoresis. (B) A SYBR Green I stained 1.1% TBE-based agarose gel is shown. (C) The autoradiogram of a 1.5% alkaline agarose gel is shown.

To address this question, we performed a transposition reaction using a supercoiled plasmid-substrate encoding a pair of *Hsmar1* terminal-inverted repeats, which are identical (for an example of such a reaction see Figure 3A below). The reaction was stopped after 15 min and treated with a restriction endonuclease that cuts at a site located asymmetrically with respect to the transposon ends (Figure 2A). Depending on whether the transposon has been cleaved at one end or the other, this generates two SEB fragments, arbitrarily designated SEB-Left and SEB-Right. We purified the fragments on a native gel and then analyzed them by denaturing gel electrophoresis after they had been 5′-end labeled (Figure 2B). Each SEB produced a pattern of three bands of similar intensities (lanes 3 and 5). These bands were of the size expected for a SEB intermediate that carries a single-strand nick at the opposite end. This most closely agrees with the constrained model (iii) in which both NTS are cleaved before the TS is cleaved at either end. This pattern could also arise in the sequential DSB model if TS cleavage at one end was immediately followed by NTS at the other end. It could also arise in the independent-cleavage model if NTS nicking was very much faster than TS cleavage. However, both alternate models are excluded by other data below (as recapitulated in the Discussion).

### End mutations reveal crosstalk

To investigate communication across the transpososome, we analyzed the effects of a mutant transposon end on the kinetics of cleavage (Figure 3). The mutant end, which we designate as ‘5G’, has an array of five GC base pairs spanning positions −2 in the flanking DNA to +3 within the transposon. This interferes strongly with cleavage. A transposon with two 5G ends (5G × 5G) reacted poorly even after several hours of incubation with transposase (Figure 3A, central panel). The nicking activity detected on the 5G end is specific: on a substrate that carries no transposon ends, nicks are undetectable.

The 5G mutation does not affect transposon binding by the transposase (data not shown). When the 5G mutation is paired with a wild-type end (5G × WT), it also does not affect transpososome assembly or the initiation of catalysis. This is evident from the kinetics of the reaction in Figure 3A. Indeed, since nicking is dependent on prior synapsis and bi-molecular synapsis is very inefficient (14), the kinetics of cleavage of the 5G × WT substrate reflects the rate of synapsis between a wild-type and a 5G end. These were identical to the kinetics of cleavage of the WT × WT substrate (Figure 3A, compare the consumption of the supercoiled substrate in leftmost and rightmost panels). However, in these reactions the transition from the nicked intermediate to the SEB product is slow and the nicked intermediate accumulates to high levels (Figure 3A, right panel). This does not fit with the independent-cleavage or sequential-cleavage models, which predict that the nicked intermediate of the reaction should be converted to the SEB intermediate at least half as fast as in the wild-type reaction.

To analyze the identity of the various cleaved termini present in these reaction mixtures, we stopped the reactions after 10 min or 3 h and digested the DNA with a restriction endonuclease. After 3′-end labeling, gel electrophoresis under native conditions revealed that the SEB intermediate produced by the 5G × WT substrate was located predominantly at the wild-type right end (Figure 3B, lanes 8 and 9). Denaturing gel electrophoresis confirmed that the 5G transposon end reacted poorly both in the 5G × 5G and 5G × WT contexts (Figure 3C, lanes 5, 6, 8 and 9). The behaviour of the WT end in the 5G × WT configuration was particularly informative: whereas the NTS was nicked rapidly, the TS was nicked very slowly (Figure 3C, lanes 8 and 9). Thus, the 5G mutations at one transposon end reduce the efficiency of TS cleavage at the WT partner end. This fits best with the constrained cleavage model in which both NTS are nicked before the first TS.

### Sequence specificity in catalysis

**Figure 4.**
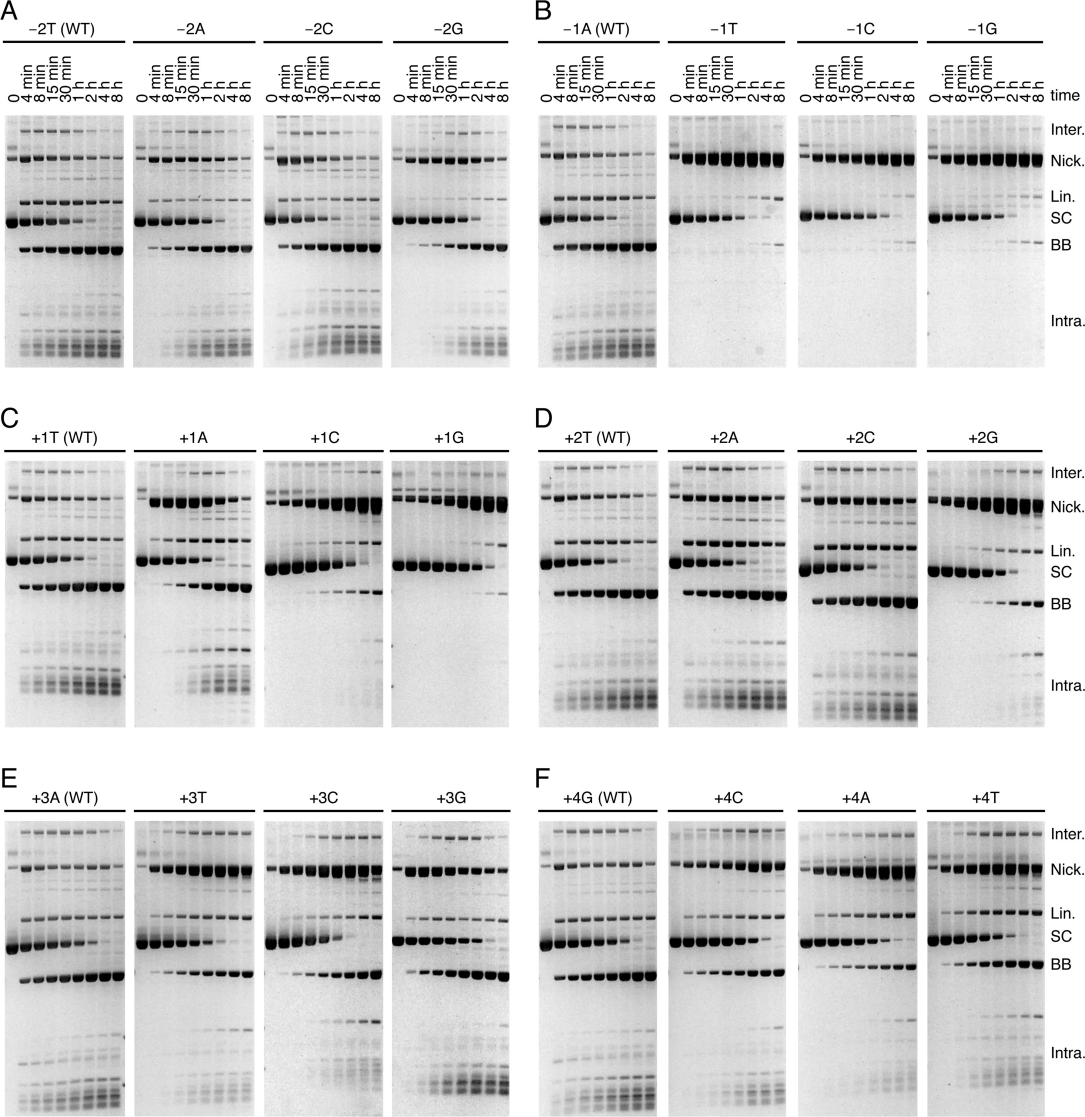
The effect of transposon-end mutations near the sites of cleavage. The kinetics of transposition reactions were analyzed with variants of a supercoiled transposon donor that carried symmetrical point mutations on both transposon ends. The nomenclature is as follows: negative numberings are for positions within the flanking DNA and positive numberings are for positions within the transposon end, as indicated in Figure 1C. A mutation is named after the nucleotide on the NTS. The products of the reactions are as illustrated in Figure 1A and described in the corresponding figure legend.

To gain further insights into the relationship between catalytic events within the transpososome, we generated a set of 18 substrates with symmetrical single base-pair mutations at both transposon ends from position −2 to +4 (Figure 4). These mutants were used to determine the contribution of sequence-specific interactions in the vicinity of the cleavage site and identify positions that affect specific catalytic steps.

*Mariner* transposons are flanked by symmetrical 5′-TA dinucleotides that arise from duplication of the target site. We previously found a mutation in the base pair directly flanking the transposon end that strongly inhibits TS cleavage (8). We extended this analysis by testing all possible double-ended mutant substrates with symmetrical nucleotide substitutions at position −2 and −1 (Figure 4A and B). All three mutations at position −2 had little effect and the kinetics of the reactions were almost as fast as with the wild-type substrate (Figure 4A). In contrast, all three mutations at position −1 were strongly defective in TS cleavage (Figure 4B). The mutant supercoiled substrates were consumed at a similar rate as the wild-type substrate demonstrating that the rates of synapsis and NTS cleavage (at least at one end) were unaffected. However, the nicked intermediate accumulated and the transition from nicked to linear and backbone, which corresponds to TS cleavage at one or two transposon ends, respectively, was very slow.

Most of the mutations at bp +1 to +4 produced less severe phenotypes than the mutations at position −1 (Figure 4C–F). Nevertheless, almost all of the mutant substrates had some level of defect in TS cleavage as can be seen from the accumulation of the nicked intermediate. Mutants +1C, +1G and +2G were the most strongly affected. These mutants also exhibited a mild defect in NTS cleavage since the consumption of the supercoiled substrate was slower than with the wild-type substrate, particularly at early time points.

Regardless of the detailed phenotypic effect of each mutation, the most striking observation is that none of the mutations greatly affect the rate of the first nick on the NTS, which is responsible for the consumption of the supercoiled substrate. This shows that the assembly of the transpososome and the NTS cleavage steps are less dependent on sequence-specific interactions between transposase and the last few nucleotides of the transposon ends than is the transition between NTS and TS cleavage.

### A structural transition between NTS and TS cleavage is coordinated at the ends

**Figure 5.**
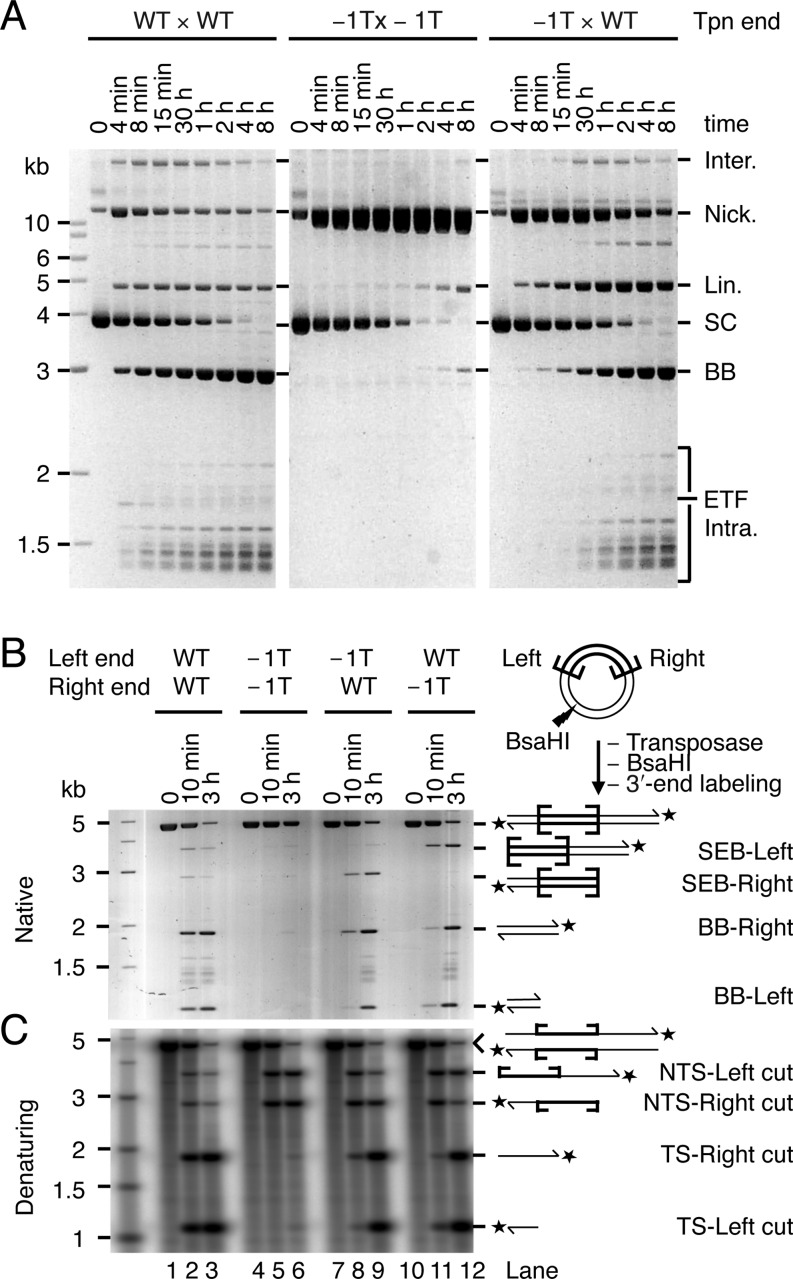
The structural transition preceding TS cleavage is coordinated at the two ends. (**A**) The kinetics of transposition reactions with a supercoiled substrate that carried two wild-type transposon ends (WT × WT), two −1T mutant transposon ends (−1T × −1T) or a −1T mutant and a wild-type end (−1T × WT) were analyzed by native agarose gel electrophoresis. The products of the reactions are as illustrated in Figure 1A and described in the corresponding figure legend. The gels with the WT × WT and −1T × −1T substrates are the same as the corresponding ones in Figure 4B. They were reproduced here to ease comparison with −1T × WT. (**B, C**) The products of transposition reactions with the substrates used in part A, plus the mutant WT × −1T substrate, were digested with the restriction enzyme BsaHI, 3′-labeled with α-^32^P-dCTP and the Klenow enzyme and analyzed by native (B) and denaturing (C) agarose gel electrophoresis. (B) A SYBR Green I stained 1.1% TBE-based agarose gel is shown. (C) The autoradiogram of a 1.5% alkaline agarose gel is shown.

The mutations at bp −1 are ideal for probing the crosstalk between transposon ends because they strongly and specifically affect TS cleavage. When a −1T mutant was paired with a wild-type end (−1T × WT), transposon excision was efficient and the backbone product accumulated to elevated levels compared to the double mutant (Figure 5A). Thus, the WT end rescued the mutant end for TS cleavage. This indicates that specific contacts with the nucleotide in −1 are important for a structural transition that is coupled between the two ends of the transposon.

To analyze the identity of the various cleaved termini present in these reactions, we digested the DNA with a restriction endonuclease. After 3′-end labeling, gel electrophoresis under native conditions confirmed that almost no double-strand cleavage products were generated with the −1T × −1T substrate (Figure 5B, lanes 5 and 6). However, with the single mutants, DSBs were produced at both the WT and the −1T ends, confirming that the WT end rescues the DSB deficiency on the mutant end (Figure 5B, lanes 8, 9, 11 and 12). Denaturing gel electrophoresis confirmed that NTS cleavage in the −1T double mutant was efficient, but that TS cleavage was very poor (Figure 5C, lanes 5 and 6). In the single mutants the NTS reacted as rapidly as in the wild type and double mutant substrates. TS cleavage was slower than in the WT × WT substrate but was detected at both WT and −1T ends (Figure 5C, lanes 8, 9, 11 and 12). The transition between the NTS and TS cleavage events is therefore accompanied by a structural change that is coordinated between the two halves of the transpososome.

### Structural determinants of coordination

The crystal structure of the *Mos1* post-cleavage intermediate has a long unstructured ‘clamp-loop’ feature extending from the catalytic core, across the dimer interface, where it interacts with the transposon end and a conserved WVPHEL amino acid motif on the opposite side of the transpososome (see Figure 7B–E) (4). The WVPHEL sequence is also in contact with a second conserved motif, YSPDL. This provides the clamp loop of one subunit with a fairly direct connection to the active site of the opposite monomer.

**Figure 6.**
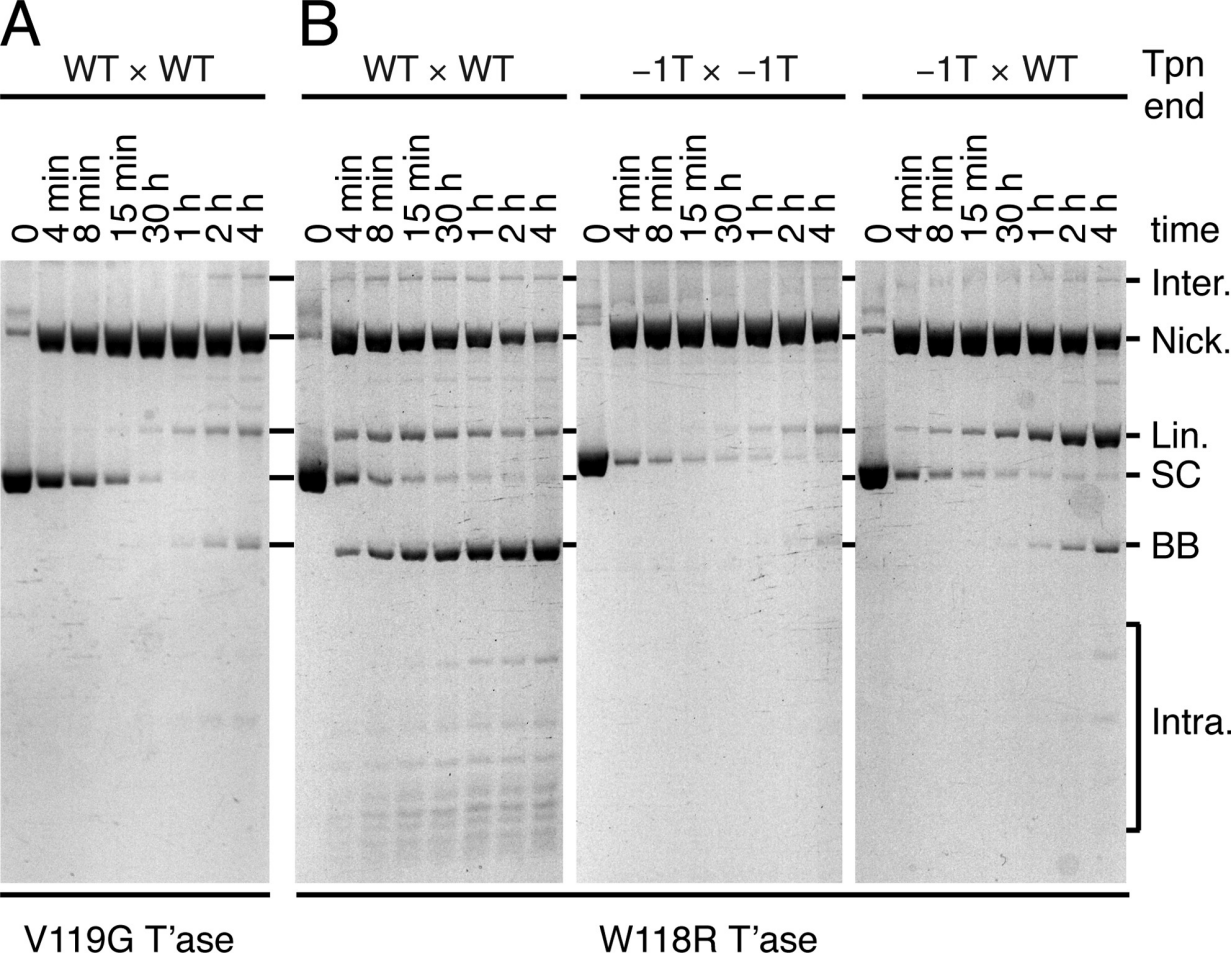
The highly conserved WVPHEL motif is involved in the coordinated transition. (**A**) The kinetics of a transposition reaction with a hypoactive transposase mutant, V119G, and a supercoiled substrate that carried two wild-type transposon ends were analyzed by native agarose gel electrophoresis. (**B**) The kinetics of transposition reactions with a hyperactive transposase mutant, W118R, and a supercoiled substrate that carried two wild-type transposon ends (WT × WT), two −1T mutant transposon ends (−1T × −1T) or a −1T mutant and a wild-type end (−1T × WT) were analyzed by native agarose gel electrophoresis. The products of the reactions are as illustrated in Figure 1A and described in the corresponding figure legend.

**Figure 7.**
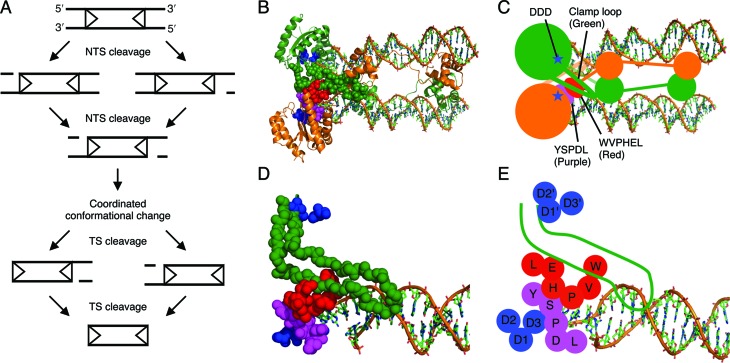
The order of catalytic events during *mariner* transposition and the structural relationship between conserved sequence motifs in the post-cleavage *Mos1* intermediate. (**A**) Transposon excision is initiated by cleavage of the NTS at the two transposon ends. This is followed by a structural change that is coordinated between the two halves of the transpososome. The transposase then cleaves the TS at the two ends, excising the transposon from the donor sequence. Transposon ends are represented as arrowheads. (**B–E**) The structural relationship between the clamp loop and the conserved sequence motifs in the crystal structure of the *Mos1* post-cleavage transpososome (4). (B, C) The *trans* architecture of the transposome. DNA is shown as sticks, transposase as ribbons with one subunit in green and the other subunit in orange. The active site (blue) and the structural features making up the interface between transposase subunits are represented as spheres. (D, E) Zoom of the interactions between the clamp loop of one subunit (green) with the conserved sequence motifs of the second subunit: WVPHEL (red), YSPDL (purple) and DDD (blue). The long unstructured loop of one subunit interacts with the conserved WVPHEL motif of the second subunit.

Many WVPHEL mutants of *Hsmar1* and *Himar1* are hyperactive in a bacterial transposition assay (27,28). It was shown with *Hsmar1* that the mutations increase the affinity of the developing transpososome for the second transposon end, which increases the rate of synapsis (28). The WVPHEL motif of *mariner* is therefore important in inter-subunit communication even before the initiation of catalysis and the generation of post-cleavage intermediate in which its *trans*-interactions with the clamp loop are observed. We therefore wondered whether these structural elements might also be involved in the conformational change that coordinates cleavage of the NTS and TS.

Within the WVPHEL motif, V119G is one of a minority of hypoactive *Hsmar1* mutants (28). *In vitro*, the V119G mutant converted the supercoiled substrate to the nicked intermediate faster than wild type (Figure 6A, compare with Figure 5A). This reflects the aforementioned increase in the rate of synapsis caused by the WVPHEL mutants. However, in contrast, the conversion of the nicked intermediate to linear and backbone products was very slow. This is similar to the effect of the −1T mutation and reflects a defect in the transition between NTS and TS cleavage. The hyperactive mutant W118R also consumed the supercoiled substrate much faster than wild-type transposase (compare Figures 5A and 6B). However, it had a much milder defect in TS cleavage and the rest of the reaction proceeded normally. We also tested the W118R transposase with mutant transposon ends (Figure 6B). With the −1T × −1T substrate the reaction was similar to wild type and the nicked intermediate accumulated (compare Figures 5A and 6B). However, with the −1T × WT substrate the wild-type transposon end failed to rescue its −1T partner efficiently (compare Figures 5A and 6B). The W118R mutant thus compromises the ability of the wild-type transposon end to drive the conformational change required for TS cleavage on the opposite side of the transpososome. In other words, the coordination mechanism of the W118R mutant is not as robust as the wild-type transposase.

## DISCUSSION

Previous studies with *Mos1* and other *mariner* elements established that the NTS is cleaved first, usually 3 bp within the transposon end. This is followed by cleavage of the TS precisely at the junction between the transposon and the flanking DNA (22,24,29–31). In *Mos1*, the DSB does not require a hairpin intermediate and is instead achieved via two sequential hydrolysis steps (22). We began by confirming the lack of a hairpin intermediate in *Hsmar1* cleavage and the order of nicks at a given end (Supplementary Figure S1). Having established the nature of the cleavage events at a given transposon end, we went on to consider whether they take place independently at opposite ends of the transposon or whether they are coordinated in some way. Three models can be envisioned. Double-strand cleavage could either (i) take place independently at each end, (ii) take place sequentially at one end before the other or (iii) have a constrained mechanism in which the NTS is cleaved at both ends before either of the TSs is cleaved (Figure 1D). Consistent with the latter model, we found that both NTS are nicked in the SEB intermediate (Figure 2B). Furthermore, in the 5G × WT transposon the unreactive mutant end prevented TS cleavage, but not NTS cleavage, at its wild-type partner end (Figure 3C, lanes 8 and 9). While this is consistent with the constrained mechanism of model iii, it contradicts the independent and sequential models. Finally, when a transposon carries symmetrical −1T mutations, which prevent TS cleavage, the NTS is cleaved efficiently at both ends (Figure 5C, lanes 5 and 6). This contradicts the sequential model. These data together demonstrate that the cleavage events in the *mariner* transpososome proceed through a constrained mechanism where the NTS must be cleaved at both ends before the TS is cleaved at either (Figure 7A).

The constrained mechanism of cleavage suggests that there must be some communication between transposase subunits to transmit signals across the transpososome. The transition between NTS and TS cleavage events requires specific interactions with the base pair directly flanking the transposon end. This was previously demonstrated by the cleavage defect of a −1G × −1G mutant transposon and was extended to the other −1 mutants here (Figure 4B and (8)). We furthermore found that a wild-type transposon end is able to rescue cleavage at a −1 mutant end (Figure 5). This demonstrates that the transition between NTS and TS cleavage is coordinated between the two sides of the complex. Communication within the transpososome therefore dictates the order of cleavage events and ensures that both NTS are cleaved before it proceeds to TS cleavage.

Our data show that the transpososome undergoes progressive conformational changes that correspond to the chemical steps of the reaction. Furthermore, the subunits on either side of the transpososome appear to be structurally and functionally coupled. Thus, we envisage that the conformational change associated with successful nicking at a wild-type transposon end helps to overcome an increase in the activation energy for nicking on the opposite side of the complex caused by the −1T mutation. In this way, a wild-type transposon end is able to rescue a mutant partner.

In the *Mos1* post-cleavage intermediate, the DNA-binding and catalytic domains of a given subunit are engaged with opposite ends of the transposon (Figure 7B, C and (4)). This defines a *trans* architecture for catalysis. The *cis* end is defined as the one engaged by the DNA-binding domain. The linker region, which connects the DNA-binding and catalytic domains and contains the conserved WVPHEL motif, makes extensive interactions with the transposon end in *trans*. In the catalytic domain, an extended ‘clamp loop’ emanates from the conserved beta sheet of the RNase H-like structural core. The loop extends across the dimer interface and interacts with the WVPHEL motif in *trans*, and the tip of the loop interacts with the *cis* transposon end (Figure 7D and E). On the opposite side from its interactions with the clamp loop, the linker interacts with the conserved YSPDL motif, which forms part of the *cis* active site. Although this extensive network of interactions is seen in the post-cleavage intermediate, at least some of the elements must be important at earlier stages of the reaction. In *Hsmar1*, many WVPHEL mutants are hyperactive (28). They perform synapsis more quickly, which indicates that they are somehow involved in the communication between subunits that lower the affinity of the developing transpososome for the second transposon end (15,28). Here, we found that the WVPHEL motif is also a critical component of the coordinated transition between NTS and TS cleavage (Figure 6). This was evident from the phenotypes of hypoactive and hyperactive WVPHEL mutants. Firstly, a transposition reaction with the hypoactive V119G mutant stalled after the first nick, indicating that the mutant is defective in the transition between NTS and TS cleavage (Figure 6A). Secondly, when the hyperactive W118R transposase was tested with the −1T × WT substrate, the wild-type transposon end was ineffective in rescuing cleavage at the −1T mutant end (compare rightmost panels of Figures 5A and 6B).

A yeast two-hybrid screen previously identified two mutations within the WVPHEL motif of *Mos1* that affect transposase dimerization: V120G and L124S (32). Since this assay did not involve transposon ends, the signal presumably reflected direct interactions between transposase monomers. This shows that the WVPHEL motif is important for dimerization at the very earliest stages of the reaction, before interactions with the transposon end(s) are established. *In vitro*, the *Mos1* V120A nicks a supercoiled substrate efficiently but the transitions to SEB and backbone products are inefficient (25). This is similar to the *Hsmar1* V119G mutant and supports a role for the WVPHEL motif in the coordinated transition that precedes TS cleavage (Figure 6C). Biochemical characterizations of the *Mos1* L124S mutant were somewhat less clear. One study reported that the mutant is essentially unreactive, with a nicking activity that was not detectably higher than the non-specific DNA degradation activity of the *Mos1* transposase (25). Another study showed that the *Mos1* L124S mutant stalls at the SEB intermediate, which suggests that the mutation affects the coordination between transposase subunits (32). In the light of our current data, the latter result could be interpreted specifically as the consequence of a defect in the coupled transition between NTS and TS cleavage.

In contrast to the ends of *Hsmar1*, which are identical, the ends of *Mos1* have four substitutions. It is unknown whether the substitutions are an adaptive feature of the system or due to genetic drift. In any case, *Mos1* transposase binds and cleaves the right end with a 5-fold and a 10-fold preference, respectively (32,33). The most relevant of the substitutions in the current context is bp+1 T to C. In *Hsmar1*, all three nucleotide substitutions at position +1 lead to a defect in TS cleavage (Figure 4C). The defect is rescued when the mutants are paired with a wild-type transposon end (Supplementary Figure S2). This suggests that a +1 mutation in *Mos1* might be due to genetic drift. Assuming so, one could view the optimal right end as wild type and the suboptimal left end as a mutant with a TS-cleavage (and DNA-binding) defect. The phenotype of the *Mos1* L124S mutant, which stalls at the SEB stage of the reaction, is therefore somewhat analogous to the activity of *Hsmar1* W118R with the −1T × WT substrate (Figure 6B). It therefore appears that the *Mos1* L124S transposase is similar to *Hsmar1* W118R and has a defect in the communication between transposase subunits during the transition that precedes TS cleavage. Thus, when the transpososome consists of a wild-type transposon end (or right end in *Mos1*) paired with a −1T mutant end (or left end in *Mos1*), the nicked and SEB intermediates accumulate because the failure of communication in the mutant transpososome prevents effective rescue of the mutant transposon end (Figure 6B).

Before the characterization of *mariner* cleavage the hairpin strategy was assumed to be the universal mechanism by which the phosphoryl transfer activity of the RNase H fold could be extended onto the opposite strand. The way in which *mariner* deals with this problem remains an intriguing mystery. All of the ideas that have been proposed require a gross reorganization of the transpososome between NTS and TS cleavage (4,8,10). The reorganization could involve subunit exchange or reorientation of a single active site to accommodate the TS, which is cleaved second. The chemistry of the RNase H family of enzymes is catalysed by the two-metal-ion mechanism (34). Although two metal ions are seen in crystals of RNase H, only one is present in the transposase structures (3–4,35). However, Nowotny et al. have speculated that, in contrast to RNase H itself, the transposase members of the RNase H family have a symmetrical transition state (35,36). In principle, this may allow a single active site to accommodate strands of opposite polarity by a simple rotation of the DNA helix about its long axis. Whichever mechanism is actually used, it is clear that a more or less significant conformational change would be required between the cleavage of opposite strands. Indeed, this probably explains the long delay observed between NTS and TS cleavage in kinetic studies of the reaction (*t*_1/2_ ≈ 30 s and 15 min for NTS and TS cleavage, respectively (8)).

Although the precise mechanism that accommodates the strands of opposite polarity remains a mystery, the present results show that it is coordinated between the two sides of the complex.

## SUPPLEMENTARY DATA

Supplementary Data are available at NAR Online.

## FUNDING

The Wellcome Trust (http//www.wellcome.ac.uk/) [WT093160 to R.C.]; BBSRC Doctoral Training Grant [C.C.B.]. Funding for open access charge: Wellcome Trust. The funders had no role in the study design, data collection and analysis, decision to publish or preparation of the manuscript.

*Conflict of interest statement*. None declared.

## Supplementary Material

SUPPLEMENTARY DATA
