# Supplementary material for: Crosstalk between transposase subunits during cleavage of the mariner transposon
Source: Nucleic Acids Res. 2014 Mar 12;42(9):5799–808. doi: 10.1093/nar/gku172 (PMC4027188; doi:10.1093/nar/gku172)
Supplement: SUPPLEMENTARY DATA [file supp_42_9_5799__index.html]

Crosstalk between transposase subunits during cleavage of the mariner transposon — SUPPLEMENTARY DATA 

# Crosstalk between transposase subunits during cleavage of the *mariner* transposon

## SUPPLEMENTARY DATA

**Files in this Data Supplement:**

- Supplemental Figures
