# Supplementary material for: Crosstalk between transposase subunits during cleavage of the mariner transposon
Source: Nucleic Acids Res. 2014 Mar 12;42(9):5799–808. doi: 10.1093/nar/gku172 (PMC4027188; doi:10.1093/nar/gku172)
Supplement: SUPPLEMENTARY DATA [file supp_gku172_Claeys_Bouuaert_Supplementary_Data_Reference_Updated.pdf]

Crosstalk between transposase subunits during cleavage of the *mariner* transposon

Corentin Claeys Bouuaert, Neil Walker, Danxu Liu and Ronald Chalmers

Supplementary Data

SUPPLEMENTARY DATA

Double strand cleavage at the *Hsmar1* transposon ends by sequential hydrolysis.

Kinetic analysis of the transposition reaction was performed with a 3'-labeled linear substrate (Supplementary Figure S1A). We also included pBluescript as target DNA. Reactions were split in two and analyzed by native and denaturing polyacrylamide gel electrophoresis (Supplementary Figure S1B, C). Under denaturing conditions, the cleaved NTS was visible after the first minute of the reaction (Supplementary Figure S1C, lane 2). Similar to the results for *Mos1*, the cleaved TS appeared slightly later, matching the kinetics of excision in the native gel.

In the hairpin reaction the order of events is constrained and the TS or the NTS must always be cleaved first or second, depending on the polarity of the reaction (see Figure 1B). However, *mariner* cleavage is not subjected to this constraint. Thus, although the kinetic analysis suggests that the NTS is cleaved first, it is possible that the mechanism is in fact unconstrained and that the NTS is simply cleaved faster on average. Evidence for a constrained order of cleavage in *Mos1* was provided by the presence of NTS nicks, but not TS nicks, in gel-purified full-length substrate at intermediate time points (22). To test this for *Hsmar1*, we purified the 211 bp 'uncleaved' fragments from the 0, 4 and 30 minutes reactions in Supplementary Figure S1B and examined them under denaturing conditions (Supplementary Figure S1D). A significant amount of cleaved NTS was present in the gel-purified fragment but no cleaved TS was detected (Supplementary Figure S1D, lane 3 and 4). We can therefore conclude that *Hsmar1* uses the same cleavage mechanism as *Mos1* and that the NTS is cleaved before the TS.

Conclusive proof that the *Mos1* reaction lacks a hairpin intermediate was provided by the precise cleavage of the TS in the absence of the required 3'-OH nucleophile on the NTS (10,22). We also recapitulated this result with *Hsmar1*

(Supplementary Figure 1E). Transposition reactions were performed with three variants of a 60 bp linear substrate that were 5'-labeled on the TS and analyzed by denaturing gel electrophoresis. The substrate either contained an intact transposon end with 20 bp double-stranded flanking DNA (substrate 1), or was pre-nicked at the site of NTS cleavage (substrate 2), or lacked the flanking NTS and therefore also lacked the 3'-OH required for the hairpin reaction (substrate 3). Transposition was efficient with all three substrates, as is evident from the consumption of the 60 nt TS and the presence of various cleavage and integration products (Supplementary Figure 1E, lanes 2, 4 and 6). This confirms that double strand cleavage at the *Hsmar1* transposon ends does not proceed through a hairpin intermediate.

Whereas the TS is nicked efficiently in the absence of the flanking NTS, the reciprocal situation introduces a severe defect (Supplementary Figure 1F). Thus, when the flanking TS is either pre-nicked or absent, the NTS fails to cleave (Supplementary Figure 1F, lanes 4 and 6). This further supports that the NTS is always cleaved before the TS in *mariner* transposition. However, the underlying mechanism of the defect is unknown, and while it may arise from structural constraints in the active site, it may also be due to a problem during transpososome assembly.

SUPPLEMENTARY FIGURE

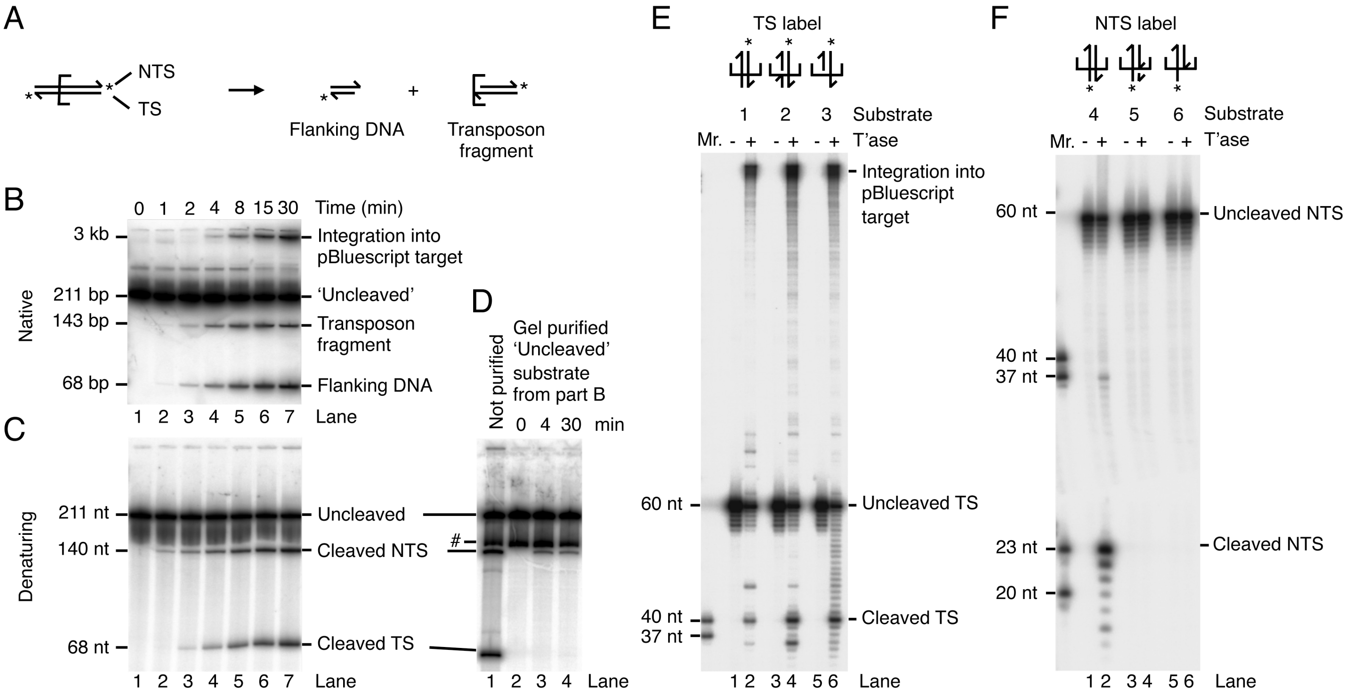

Supplementary Figure S1 : *Hsmar1* conforms to the mechanism of cleavage of *Mos1.*

A, Cleavage assay using short linear substrates. A DNA cleavage assay was performed with a 211 bp linear substrate that carried a single transposon end and was 3'-labeled at both extremities (asterisks). Double strand cleavage liberates a 143 bp linear 'transposon fragment' and a 68 bp DNA flank. Bimolecular integration of two transposon fragments within a supercoiled plasmid provided as a target generates a 3 kb linear product.

B, Kinetic analysis of DNA cleavage by native gel electrophoresis. The kinetics of strand cleavages were analyzed in a transposition reaction with the 211-bp linear substrate. A 50 µl transposition reaction contained 150 nM substrate, 150 nM transposase and 10 µg pBluescript target DNA in a standard transposition buffer. 5 µl aliquots were withdrawn at the indicted time points. The reactions were stopped and deproteinated by phenol extraction. DNA was ethanol precipitated, resuspended in TE buffer, and one tenth of each sample was analyzed on a 10 % native polyacrylamide gel.

C, Kinetic analysis of DNA cleavage by denaturing gel electrophoresis. The products of the cleavage assay shown in panel B were analyzed on a 10 % denaturing polyacrylamide gel. Two cleavage products accumulated which are the 140 nucleotides (nt) cleaved non-transferred strand (NTS) and the 68 nt DNA flank liberated upon cleavage of the transferred strand (TS).

Since the transposon is labeled on the NTS the integration product is not visible on the denaturing gel.

D, Detection of single-strand nicks. The full-length ('uncleaved') product from part B (time points: 0, 4 and 30 min) was eluted from the gel and re-loaded on a 10 % denaturing gel to assay for single strand nicking. #, An artifact of the assay.

E, F, Flanking DNA requirements in TS and NTS cleavage. Different substrates were prepared by annealing oligonucleotides, tested in a cleavage assay, and the products were analyzed by denaturing gel electrophoresis. The substrates were variants of a 60 bp linear duplex that carries a transposon end and 20 bp flanking DNA. The position of the radioactive phosphate is marked with an asterisk. Each substrate contained a full-length 5'-labeled strand (60 nt). The opposite unlabeled strand was full length, or contained a nick at the site of cleavage, or lacked the flanking strand. A 20 µl reaction contained 300 nM substrate, 200 nM transposase and 1 µg of pBluescript DNA in a standard transposition buffer. Reactions were carried out for 2 h, DNA was deproteinated and analyzed on a 10 % denaturing polyacrylamide gel. The markers (Mr.) are 5'-labeled oligonucleotides of known size that were used to generate the different substrates.

E, Double-strand cleavage in *Hsmar1* does not involve a DNA hairpin intermediate. Three substrates labeled on the TS were tested in a cleavage assay. Substrates were (1) the full-length substrate; (2) the substrate carrying a nick on the NTS and (3) the substrate lacking the NTS flanking DNA. Precise TS cleavage generates a 40 nt strand that further integrates into the supercoiled plasmid which was provided in the reaction as a target.

F, NTS cleavage requires an intact TS. Three substrates labeled on the NTS were tested in a cleavage assay. Substrates were (4) the full-length substrate; (5) the substrate carrying a nick on the TS and (6) the substrate lacking the TS flanking DNA. The main site of NTS cleavage is recessed three bases within the transposon end and produces a 23 nt labeled strand.

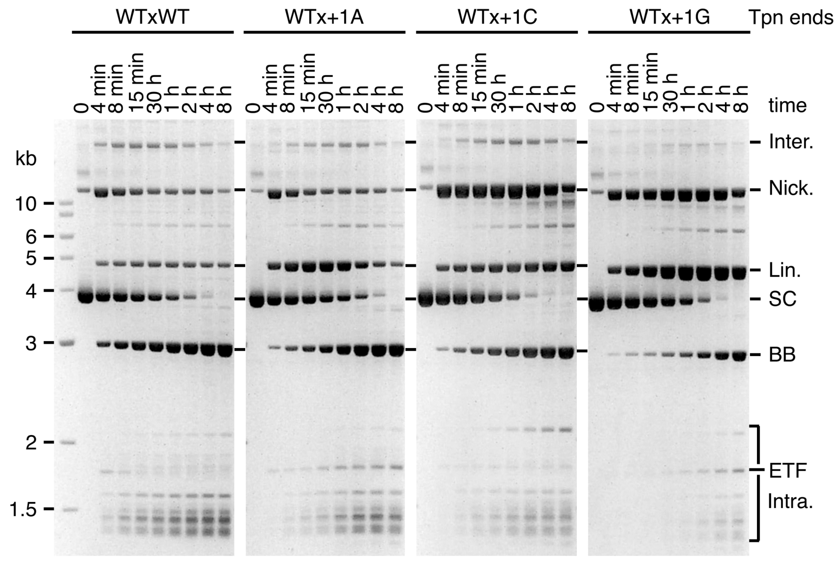

Supplementary Figure S2 : A wild-type transposon end rescues cleavage at a +1C and a +1G mutant end.

The kinetics of transposition reactions were analyzed with supercoiled substrates that carried a wild-type transposon end and a transposon end mutated at position +1. Photographs of ethidium bromide-stained 1.1 % TBE-agarose gels are shown. Substrates that carry symmetrical +1C or +1G mutant ends are strongly affected for TS cleavage (see the accumulation of nicked intermediate in Figure 4C). However, when paired with a wild-type end, the +1C or +1G ends are rescued for TS cleavage, which is evident from the accumulation of the plasmid backbone.

SUPPLEMENTARY EXPERIMENTAL PROCEDURES

Cleavage assays with short linear substrates

Cleavage assays with linear substrates were performed at elevated substrate and transposase concentrations (150-300 nM, as indicated) to promote bimolecular synapsis (14). Plasmid pRC1399 has a single 30 bp *Hsmar1* end (with flanking TA) cloned into the pBluescript polylinker and was digested with BssHII and 3'-labeled on both strands with α-^{32}P-dCTP and the Klenow enzyme. This produced the 211-bp linear substrate used in Figure 2A-D. Substrates to assay the effect of the flanking DNA (Figure 2E, F) were prepared by annealing complementary oligonucleotides. The substrate were variants of a 60 bp substrate with a transposon end and 20 bp flanking DNA, including a TA dinucleotide. The oligonucleotides used to prepare these substrates were the following (with nucleotide sequences between brackets): WT-NTS60 (TTCATTAATGCAGAATTCTATTAGGTTGGTGCAAAAGTAATTGCGGTTTTGG ATCCCTGT); WT-NTS37 (GGTTGGTGCAAAAGTAATTGCGGTTTTGGATCCCTGT); WT-NTS23 (TTCATTAATGCAGAATTCTATTA); WT-TS60 (ACAGGGATCCAAAACCGCAATTACTTTTGCACCAACCTAATAGAATTCTGCA TTAATGAA); WT-TS40 (ACAGGGATCCAAAACCGCAATTACTTTTGCACCAACCTAA); WT-TS20 (TAGAATTCTGCATTAATGAA). To assess the flanking DNA requirements for TS cleavage, three substrates 5'-labeled on the TS were prepared: a full-length substrate (oligos WT-NTS60 and WT-TS60), a substrate nicked at the site of first-strand cleavage (3 bases within the transposon end) (oligos WT-NTS23, WT-NTS37 and WT-TS60) and a substrate lacking the 23 bp of flanking DNA on the NTS (oligos WT-NTS37 and WT-TS60). To assess the flanking DNA requirements for NTS cleavage, three substrates 5'-labeled on the NTS were prepared: a full-length substrate (oligos WT-NTS60 and WT-TS60), a substrate nicked at the site of second-strand cleavage (oligos WT-NTS60, WT-TS40 and

WT-TS20) and a substrate lacking the 20 bp flanking DNA on the TS (oligos WT-NTS60 and WT-TS40). DNA duplexes that mimicked a pre-nicked substrate carried a 5'-phosphate on the nicked strand that was introduced on the oligonucleotide by PNK in Ligase buffer. Cleavage products were analyzed on a 10 % Tris-acetate-EDTA polyacrylamide gel (native) or a 10 % Urea-Tris-borate-EDTA polyacrylamide gel (denaturing).
